# 

**Published:** 1855-04

**Authors:** 


### CONTENTS OF THIS (APRIL) NUMBER.

ORIGINAL COMMUNICATIONS.

Address of W. H. Goddard, Delivered before the Miss. Val. Ass. of Dental Surgeons, Feb. 33, 1855 .... 155
Alloys. By Geogge Watt, M.D., D.D.S., Read before the Miss. Val. As3. Feb. 23d 1855 .... 170
Preparation of Cavities for filling teeth. By A. S. Talbert, D.D.S. Lexington, Ky. Delivered before the Miss. Val. Ass. of Dental Surgeons, Feb. 23, 1855 .... 175

PROCEEDINGS OF SOCIETIES.

Eleveverth Annusl Meeting of the Miss. Val. Ass. of Dental Surgery .... 183
Minutes of the third Annual Meeting of the Ohio Dental College Association .... 192
Valedictory Address to the Graduating Class. By Dr. James Taylor, Delivered Feb. 23d, 1855 .... 199
Munro's Reply ....
Harris on Air Chamber .... 216

SELECTED ARTICLES.

Method of avoiding the warping or springing of metallic Plates in the process of Solderiug full sets of Teeth .... 218
A new method for operating upon loose or Sensitive Teeth. By W. A. Helme, Dentist, Providence, R. I. .... 220
The Dental Profession in Europe .... 222
Gutta Percha Bases for Artificial Teeth .... 225
Editorial, .... 228

